# Knowledge, Attitudes, and Practices of COVID-19 Vaccination among Adults in Singapore: A Cross-Sectional Study

**DOI:** 10.4269/ajtmh.21-1259

**Published:** 2022-07-18

**Authors:** Joseph Yuen Juin Cheng, Shaun Seh Ern Loong, Clare Elisabeth Si Min Ho, Kai Jing Ng, Miki Min Qi Ng, Ryan Choon Hoe Chee, Tiffany Xuan Ling Chin, Francis Jia Yi Fong, Song Ling Germain Goh, Kumaresh Natarajan S/O Venkatesh, Zi Ying Sim, Zach Yung Shen Chan, Shayne Pek, Xin Wei Liew, Yan Qing Cherie Ong, Benjamin Wu, Luke Yu Xuan Yeo, Tony De Rong Ng, Celeste Zi Hui Ng, Wei Wen Soon, Bryan Yichong Shi, Ruth Si Man Wong, Sean Tan, Ivan Leong, Celeste Li-Lynn Chan, Jia Wen Tan, Junxiong Pang

**Affiliations:** ^1^ Yong Loo Lin School of Medicine, National University of Singapore and National University Health System, Singapore;; ^2^ Saw Swee Hock School of Public Health, National University of Singapore and National University Health System, Singapore

## Abstract

Public health measures promoting compliance of COVID-19 vaccination requires understanding of knowledge, attitudes, and practices (KAP). This study explored the KAP and risk factors influencing COVID-19 vaccination, including changes in preventive practices before and after vaccination in a high-income country, Singapore. An online cross-sectional study among Singaporeans and permanent residents aged 21 years and older was conducted from July to August 2021. Univariate and multivariable logistic regressions using RStudio version 1.4.1106 was performed to assess associations between demographic factors, KAP, and vaccination status. *P* values < 0.05 were considered statistically significant. A total of 869 respondents completed the survey. Individuals with higher knowledge (adjusted odds ratio [aOR] = 2.00, *P* = 0.024), perceived efficacy (aOR = 1.19, *P* = 0.004), perceived safety (aOR = 1.20, *P* = 0.005), and willingness to uptake (aOR = 1.55, *P* < 0.001) scores were more likely to be vaccinated. There was a significant increase in the use of proper handwashing techniques among the vaccinated group before and after vaccinations. The governmental risk communication approaches have been useful in instilling high levels of vaccine knowledge. High levels of good attitudes about and knowledge of COVID-19 vaccination were associated with a high level of vaccination practices. Good perceived vaccine efficacy and confidence in government were also associated with positive vaccine uptake. This study paves the way for more targeted government measures to be implemented to improve vaccination rates of COVID-19 booster vaccines in a high-income country like Singapore.

## INTRODUCTION

### SARS-CoV-2.

The novel SARS-CoV-2 was declared a global pandemic on March 11, 2020, and a public health emergency of international concern.^1^ Since the first imported case of COVID-19 on January 23, 2020, Singapore saw a total of approximately 235,480 recorded cases and 576 deaths as of November 13, 2021,^2^ with case fatality rates of 0.244%. These have far exceeded the 238 recorded cases and 33 deaths of severe acute respiratory syndrome (SARS) outbreak in Singapore back in 2003.^3^

### Vaccinations.

Globally, the WHO, governmental bodies, and pharmaceutical companies collaborated to expedite trials to produce a safe and effective vaccine at a revolutionary pace. The accelerated speed of vaccine development was essential due to the highly infective nature of SARS-CoV-2.^4^ Guided by the experience of SARS, the Ministry of Health (MOH) of Singapore orchestrated a detailed approach to combat the current pandemic. Currently, COVID-19 vaccination has been made free for Singaporeans and permanent residents aged 12 years and older to increase accessibility and equity of this public health good. To date, there are three vaccines approved for use by Health Sciences Authority Singapore: Pfizer-BioNTech, Moderna, and Sinovac. As of November 12, 2021, 85% of the Singaporean population had completed their full vaccination regimen.^5^

### Vaccine hesitancy and herd immunity.

Nevertheless, vaccine availability, accessibility, and efficacy do not guarantee successful high coverage and uptake of vaccination due to the phenomenon of vaccine hesitancy.^6^ This phenomenon is especially relevant given concerns regarding the perceived safety of a rapidly developed vaccine, even in high-income countries. Overcoming vaccine hesitancy is important to achieve a sustained level of herd immunity in an attempt to protect the most vulnerable groups who are unable to receive or sufficiently respond to the vaccine.^7^ A study conducted in London concluded that contextual influences (communication, media, historical, politics, religion), individual and group influences (experience with past vaccinations, knowledge, trust in health systems), and vaccine-specific issues (risk and benefit, cost, design of vaccination programs) were among the key determinants in choosing to accept, reject, or delay vaccination.^8^ General vaccine hesitancy may be governed by factors unique to each country.

### Knowledge, attitudes, and practices model.

Knowledge, attitudes, and practices (KAP) factors based on the Health Belief Model are commonly used to quantify individual belief profiles in public health measures.^9^ Studies have been conducted by individual countries to understand the KAP factors within the local population to customize effective public health efforts in COVID-19.^10^ An Oman-based study identified education levels, source of vaccine knowledge, and history of chronic disease as key factors influencing vaccine uptake in their community.^11^ Another study conducted in Bangladesh determined that their study population had poor knowledge but positive attitudes toward the vaccine and as such recommended immediate health education programs to bolster uptake.^12^ In high-income countries, a study done in the United Kingdom found that individuals with an intention to vaccinate had greater knowledge and a more positive attitude toward the COVID-19 vaccine.^13^ An Italian study found that intention to get vaccinated increased with previous flu vaccine behavior but decreased with doubts about vaccines.^14^ Looking into the future, as more high-income countries like Singapore gradually move from COVID-19 pandemic to endemic, a good understanding of KAP factors relevant to high-income country context will be essential in this long-term strategy to sustain herd immunity.^15^

### Attitudes toward vaccine safety.

Attitudes toward vaccine safety are a significant factor that contributes to vaccine hesitation. A study in Finland showed that the strongest predictor of COVID-19 vaccinations was the perceived safety of the vaccine.^16^ However, due to expedited vaccine trials, long-term effects of the vaccines have yet to be comprehensively documented. Existing literature has suggested that in the event of a lack of information about the safety of a new vaccine, a person’s experience and attitudes toward other existing vaccines strongly shape their current vaccine decisions. This was seen in a study conducted across three countries^17^ and in an Australian study,^18^ both of which showed that the uptake of seasonal influenza vaccinations was a strong positive determinant in the uptake of pandemic vaccinations. On the other hand, other existing vaccines such as the Pandemrix vaccine for swine flu may negatively influence opinions toward COVID-19 vaccines because it has been associated with a higher risk of narcolepsy, which may have exacerbated vaccine hesitancy.

Furthermore, studies showed that psychological stress is another factor that influences people’s attitudes on the safety of the vaccine. Increased stress decreases immune system response and, as a result, vaccine efficacy after administration.^19^ A study from Brazil demonstrated that anxiety elicits immunization anxiety-related reactions post-immunization,^20^ decreasing the willingness for vaccination among people who have psychological reservations toward the vaccine for fear of adverse effects. Furthermore, a study conducted in the United Kingdom and Ireland found that the psychological processes that affect vaccine hesitancy are reflective of the fundamental “attitude roots” model of science rejection.^21^

There are limited studies that focus on high-income countries—in particular, within Asia—and thus, this paper study used the KAP framework to determine the factors that influence Singaporean adults’ COVID-19 vaccination status.

## MATERIALS AND METHODS

### Ethical approval and confidentiality of data.

Ethics approval was granted by the National University of Singapore Department Ethics Review Committee (SSHSPH-142). Informed consent was sought from respondents on the introductory web page before participation in the study. Confidentiality of data was assured as participants were informed that their responses would remain anonymous, and no personal identifiers would be included. Only completed survey data were included in the study and formed the research sample.

### Study design and questionnaire development.

A cross-sectional study was conducted with convenience sampling using an online survey from July 2021 through August 2021 because onsite face-to-face survey was not feasible due to national COVID-19 restrictions in Singapore. The inclusion criteria were Singaporeans and permanent residents, aged 21 and older. Interested respondents who responded as either being non-Singaporean or below age 21 were excluded from the survey. The questionnaire was created in English using REDCap software, and a shareable link was disseminated publicly on social media platforms, including WhatsApp, Telegram, Instagram, and Facebook. The questionnaire was divided into five main sections: socioeconomic demographics, knowledge, attitudes, practices, and symptoms or adverse effects of the vaccine. All questions related to KAP were adapted from the Health Belief Model, similar studies conducted by other countries on their local population,^12^^,^^22^^–^^24^ and information from the Singapore MOH website detailing the latest updates on the COVID-19 vaccination policy in Singapore.^5^ For this study, responses were only considered complete if the demographic section and more than 95% of the total survey was completed.

The questionnaire followed guidelines suggested by Tsang et al.^25^ The dimensionality, format of administration and item formats were consistent with questionnaires from previous studies ensuring validity.^12^^,^^22^^–^^24^ Using verified and updated sources of information before launching ensured the questionnaire’s level of relevance.

### Socioeconomic demographics.

Basic demographic data collected were age, race, sex, marital status, highest level of education, monthly family income, and type of employment. Participants were also asked whether they had received the COVID-19 vaccine, whether they take the flu vaccine on a yearly basis, if they had any medical conditions, and if they were required to undergo rostered routine testing (RRT).

### Knowledge, attitudes, and practices.

To assess the KAP level of the respondents, 40 to 41 items were included in the questionnaire. The knowledge section consisted of 14 items with three options each (“yes,” “no,” and “not sure”). These items were chosen to evaluate participants’ understanding of the availability, effectiveness, and suitability of COVID-19 vaccines in Singapore. The attitudes section comprised 12 items, with responses indicated on a 5-point Likert scale (1 = strongly disagree, 2 = disagree, 3 = neutral, 4 = agree, 5 = strongly agree). These items gathered participants’ views on COVID-19 vaccine efficacy and safety and respondents’ willingness to receive the vaccine. The practices section contained 14 to 15 items in total. A fixed set of six items (“yes” or “no” questions) was used to assess individual’s behavior before—and, if applicable, after—receiving the COVID-19 vaccine. Depending on whether participants had received the COVID-19 vaccine, another set of two or three items was included to determine the reasons for their decision.

### Symptoms or adverse effects.

An additional section, comprising eight items (“yes” or “no” questions), asked participants whether they have experienced certain symptoms over the past 14 days (if they have not received the vaccine) or within 14 days of vaccination.

## DATA ANALYSIS

### Overall data analysis strategy.

Data analysis was carried out using RStudio (version 1.4.1106; Boston, MA). The data analysis design focused on the differences between COVID-19 vaccinated and unvaccinated groups, as well as associated risk factors. The vaccinated group refers to participants who had both doses of the vaccine or have already booked their appointment for either the first or second dose. The factors of interest include demographic data such as age, race, sex, marital status, household income, education level, employment sector, medical conditions, annual flu vaccine uptake, RRT status, and vaccination status.

### Scoring method of KAP domains.

Original data collected from the survey were coded (e.g., “no” and “yes” as 0s and 1s) during the data cleaning stage to facilitate analysis. Each of the KAP domains were given aggregate scores based on the sum of responses to the relevant items. As such, knowledge was scored between –14 and 14, and practices between 1 and 6. The attitudes domains were divided into three scores owing to their thematic differences, and thus the perceived efficacy, perceived safety, and willingness to uptake scores were derived.

### Univariate analysis.

Univariate analysis of the correlation was performed between participants’ KAP and COVID-19 vaccination status. Independent *t* test was used for binary demographics. One-way analysis of variance and chi-square test were used for categorical factors and linear regression for continuous variables. Univariate logistic or linear regression was then performed to identify correlation. A *P* value < 0.05 was considered statistically significant. Factors with significant differences were then identified and used in multivariable analysis as potential confounders.

### Multivariable analysis.

Multivariable analysis was performed using a multivariable logistic or linear regression model dependent on the datatype of the dependent variable to quantify the relationship between participants’ demographics, KAP sections, and the aggregate measures for each section. Adjusted odds ratio (aOR) or standardized coefficients were derived to assess independent risk factors associated with the vaccination group.

## RESULTS

### Respondents’ overall sociodemographic characteristics.

A total of 869 respondents completed the survey. The mean participant age was 41.5 (± 15.63) years, with the majority of participants in the age range of 21 to 29 years (33.7%; *N* = 293) and 50 to 59 years (31.5%; *n* = 274). Furthermore, 93.4% (*n* = 812) were Chinese, 60% (*n* = 521) were female, and 57% (*n* = 495) were married; 65.5% (*N* = 569) had an education at the university level or higher, and 51.8% (*N* = 450) had monthly household income levels greater than $10,000. Flu vaccine was received annually by 26.8% (*n* = 233) of participants, and 16.2% (*N* = 141) of participants had at least one medical condition, with 7.6% (*N* = 66) having cardiovascular conditions; 14.2% (*N* = 123) of participants were on RRT. Under sectors of employment, 38.3% (*N* = 333) were either students or unemployed, and the second largest group was the finance and business sector (16.1%; *n* = 140) (Table 1).

**Table 1:** Sociodemographic characteristics of respondents (*N* = 869)

	Total *n* (%), *N* = 869	National statistics %*	Vaccinated group† *n* (%), *N* = 823	Unvaccinated group‡ *n* (%), *N* = 46	*P* value
Age (years) (mean ± SD)	41.49 ± 15.63	41.5	41.43 ± 5.73	42.56 ± 13.68	0.589
Age groups					0.110
21–29	293 (33.71%)	16.09	282 (34.26%)	11 (23.91%)	
30–39	71 (8.17%)	18.42	64 (7.78%)	7 (15.22%)	
40–49	144 (16.57%)	18.50	132 (16.04%)	12 (26.09%)	
50–59	274 (31.53%)	18.23	262 (31.83%)	12 (26.09%)	
≥60	87 (10.01%)	28.76	83 (10.09%)	4 (8.70%)	
Race					0.341
Chinese	812 (93.44%)	75.99	768 (93.32%)	44 (95.65%)	
Malay	13 (1.50%)	12.51	12 (1.46%)	1 (2.17%)	
Indian	24 (2.76%)	8.48	24 (2.92%)	0 (0.00%)	
Eurasian	5 (0.58%)	0.38	4 (0.49%)	1 (2.17%)	
Others	15 (1.72%)	2.64	15 (1.82%)	0 (0.00%)	
Sex					0.290
Female	521 (59.95%)	51.63	490 (59.54%)	31 (67.39%)	
Male	348 (40.04%)	48.39	333 (40.46%)	15 (32.61%)	
Marital status					0.076
Married	495 (56.96%)	62.33	463 (56.26%)	32 (69.57%)	
Unmarried	374 (43.04%)	48.39	360 (43.74%)	14 (30.43%)	
Education level					0.546
None	1 (0.12%)	10.70	1 (0.12%)	0 (0.00%)	
Primary	1 (0.12%)	13.81	1 (0.12%)	0 (0.00%)	
Secondary	37 (4.26%)	16.04	37 (4.50%)	0 (0.00%)	
Pre-University	231 (26.58%)	27.08	222 (26.97%)	9 (19.57%)	
University and higher	569 (65.48%)	32.36	534 (64.88%)	35 (76.09%)	
Others	30 (3.45%)		28 (3.40%)	2 (4.35%)	
Income level ($)					0.915
< 1,000	41 (4.72%)	35.22	39 (4.74%)	2 (4.35%)	
1,000–4,000	94 (10.82%)	25.68	88 (10.69%)	6 (13.04%)	
4,000–7,000	127 (14.61%)	17.46	120 (14.58%)	7 (15.22%)	
7,000–10,000	157 (18.07%)	9.19	147 (17.86%)	10 (21.74%)	
	450 (51.78%)	12.45	429 (52.13%)	21 (45.65%)	
Flu vaccine					**0.030**
No	636 (73.19%)	82.60	596 (72.42%)	40 (86.96%)	
Yes	233 (26.81%)	17.40	227 (27.58%)	6 (13.04%)	
Medical condition					
None	728 (83.77%)		694 (84.33%)	34 (73.91%)	0.062
≥ 1	141 (16.23%)		129 (15.67%)	12 (26.09%)	
CVD	66 (7.59%)		64 (7.78%)	2 (4.35%)	0.393
Metabolic	11 (1.27%)		11 (1.34%)	0 (0.00%)	0.430
Hypersensitivity	37 (4.26%)		33 (4.01%)	4 (8.70%)	0.126
Neoplastic	10 (1.15%)		7 (0.85%)	3 (6.52%)	**< 0.001**
Other	40 (4.60%)		36 (4.37%)	4 (8.70%)	0.174
Employment sector					0.216
Commerce (retail and trade)	27 (3.11%)		25 (3.04%)	2 (4.35%)	
Community, social and personal services	50 (5.75%)		46 (5.59%)	4 (8.70%)	
Education	63 (7.25%)		62 (7.53%)	1 (2.17%)	
Finance and business	140 (16.11%)		128 (15.55%)	12 (26.09%)	
Food and beverages	12 (1.38%)		11 (1.34%)	1 (2.17%)	
Hotels and tourism	4 (0.46%)		3 (0.36%)	1 (2.17%)	
STEM and healthcare	96 (11.05%)		93 (11.30%)	3 (6.52%)	
Transport, storage and communication	35 (4.03%)		32 (3.89%)	3 (6.52%)	
Unemployed (including students)	333 (38.32%)		320 (38.88%)	13 (28.26%)	
Others	109 (12.54%)		103 (12.52%)	6 (13.04%)	
RRT status					0.275
Negative	746 (85.85%)	83.03	704 (85.54%)	42 (91.30%)	
Positive	123 (14.15%)	16.97	119 (14.46%)	4 (8.70%)	
Survey subsections					
Knowledge score§ (out of a full score of 14)					
Total (mean ± SD)		5.96 ± 3.19	6.03 ± 3.17	4.67 ± 3.39	**0.011**
High/low category					**0.013**
Low		287 (33.03%)	264 (32.20%)	23 (50.00%)	
High		579 (66.63%)	556 (67.80%)	23 (50.00%)	
Attitudes scores					
Efficacy score (mean ± SD)		11.60 ± 3.23	11.68 ± 3.22	10.17 ± 3.00	**0.002**
Safety score (mean ± SD)		15.67 ± 2.74	15.85 ± 2.57	12.35 ± 3.54	**< 0.001**
Uptake score (mean ± SD)		11.37 ± 2.00	11.52 ± 1.86	8.76 ± 2.57	**< 0.001**
Practices (before) score					
Total (mean ± SD)		4.67 ± 1.22	4.67 ± 1.20	4.63 ± 1.45	0.863
High/low category					0.858
Low		332 (38.20%)	315 (38.27%)	17 (35.96%)	
High		537 (61.80%)	508 (61.72%)	29 (63.04%)	
Intersection correlation					
Sections		Coefficient	*P* value		
Attitudes–practices (after)		−0.130	**< 0.01**		
Attitude–practices (before)		−0.095	**< 0.01**		
Knowledge–attitudes		0.132	**< 0.01**		
Knowledge–practices (before)		0.020	0.57		
Knowledge—practices (after)		0.002	0.96		

RRT = rostered routine test; STEM = science, technology, engineering, or medicine. Bold *P*-values represent statistical significant variable.

* Based on National Demographics data from Singapore Census Population 2020, other than the flu vaccine status, which was taken from the National Population Health Survey 2019,^62^ and the number of people going for RRT, which was taken from the Ministry of Health website.^63^ Data for the employment status and medical conditions is unavailable.

† Vaccinated = received vaccine or waiting to receive it.

‡ Unvaccinated = has not signed up for vaccine.

§ Three responses were removed when tabulating knowledge score because respondents did not answer all of the knowledge questions.

### Key differences in characteristics between vaccinated and nonvaccinated groups.

There were 823 participants (94.7%) in the vaccinated group, and 46 participants (5.3%) in the nonvaccinated group. Among the vaccinated group, the mean age was 41.4 years (±5.73), 40.5% were male, a majority of 34.3% was in the 21- to 29-year age group, and 64.9% was educated at the university level or higher. Among the unvaccinated group, the mean age was 42.6 years (±13.68), 32.6% were male, a majority of 26.09% was in the 40- to 49-year and 50- to 59-year age groups, and a majority of 76.1% was educated at the university level or higher (Table 1). The only significant differences between the two groups were annual flu vaccine uptake (vaccinated group = 27.6%, nonvaccinated group = 13.0%, *P* value = 0.030) and presence of neoplastic medical conditions (vaccinated group = 0.85%, nonvaccinated = 6.52%, *P* value < 0.001) (Table 1).

### Demographic determinants of vaccination status.

Significant demographic determinants of positive COVID-19 vaccination status include age group 30 to 39 years (aOR = 0.34, 95% confidence interval [CI]: 0.13–0.97, *P* = 0.035), 40 to 49 years (aOR = 0.41, 95% CI: 0.17, 0.96, *P* = 0.049), and annual flu vaccine recipients (aOR = 2.64, 95% CI: 1.18–7.02, *P* = 0.030) (Table 2).

**Table 2:** Univariate and multivariate logistic regression for demographic determinants of Vaccination status

	Vaccinated *n* (%) *N* = 823	Unvaccinated *n* (%) *N* = 46	uOR (95% CI)	*P* value	aOR* (95% CI)	*P* value
Age						
21–29	282 (96.25%)	11 (3.75%)		Ref		Ref
30–39	64 (90.14%)	7 (9.86%)	0.36 (0.14,1.00)	**0.040**	0.34 (0.13–0.97)	**0.035**
40–49	132 (91.67%)	12 (8.33%)	0.43 (0.18–1.00)	**0.049**	0.41 (0.17–0.96)	**0.040**
50–59	262 (95.62%)	12 (4.38%)	0.85 (0.36–1.98)	0.706	0.84 (0.35–1.95)	0.679
≥ 60	83 (95.40%)	4 (4.60%)	0.81 (0.27–2.98)	0.723	0.76 (0.25–2.81)	0.648
Race						
Chinese	768 (94.58%)	44 (5.42%)		Ref		
Malay	4 (80.00%)	1 (20.00%)	NA	0.722		
Indian	24 (100.00%)	0 (0.00%)	NA	0.985		
Eurasian	12 (92.31%)	1 (7.69%)	NA	0.192		
Others	15 (100.00%)	0 (0.00%)	NA	0.989		
Sex						
Female	490 (94.05%)	31 (5.95%)		Ref		
Male	333 (95.69%)	15 (4.31%)	1.49 (0.76–2.71)	0.292		
Marital status						
Married	463 (93.54%)	32 (6.46%)		Ref		
Unmarried	360 (96.26%)	14 (3.74%)	1.78 (0.95–3.48)	0.080		
Education level						
None	1 (100.00%)	0 (0.00%)		Ref		
Primary	1 (100.00%)	0 (0.00%)	NA	1		
Secondary	37 (100.00%)	0 (0.00%)	NA	1		
Pre-University	222 (96.10%)	9 (3.90%)	NA	0.997		
University and above	534 (93.85%)	35 (6.15%)	NA	0.997		
Others	28 (93.33%)	2 (6.67%)	NA	0.997		
Income level ($)						
< 1,000	39 (95.12%)	2 (4.88%)		Ref		
1,000–4,000	88 (93.62%)	6 (6.38%)	0.75 (0.11–3.43)	0.734		
4,000–7,000	120 (94.49%)	7 (5.51%)	0.88 (0.13–3.82)	0.876		
7,000–10,000	147 (93.63%)	10 (6.37%)	0.75 (0.11–3.01)	0.722		
	429 (95.33%)	21 (4.67%)	1.05 (0.16–3.76)	0.951		
Flu vaccine						
No	596 (93.71%)	40 (6.29%)		Ref		Ref
Yes	227 (97.42%)	6 (2.58%)	2.54 (1.14–6.74)	**0.036**	2.64 (1.18–7.02)	**0.030**
Medical conditions						
None	694 (95.33%)	34 (4.67%)		Ref		
≥ 1	129 (91.49%)	12 (8.51%)	0.53 (0.27–1.08)	0.066		
Employment sector						
Commerce (retail and trade)	25 (92.59%)	2 (7.41%)		Ref		
Community—social and personal services	46 (92.00%)	4 (8.00%)	0.92 (0.12–5.06)	0.926		
Education	62 (98.41%)	1 (1.59%)	4.96 (0.46–109.58)	0.199		
Finance and business	128 (91.43%)	12 (8.57%)	0.85 (0.13–3.39)	0.842		
Food and beverages	11 (91.67%)	1 (8.33%)	0.88 (0.08–20.11)	0.920		
Hotels and tourism	3 (75%)	1 (25.00%)	0.24 (0.02–6.06)	0.297		
Others	103 (94.50)	6 (5.50%)	1.37 (0.19–6.38)	0.708		
STEM and healthcare	93 (96.88)	3 (3.12%)	2.48 (0.31–15.76)	0.334		
Transport, storage, and communications	32 (91.43)	3 (8.57%)	0.85 (0.11–5.53)	0.868		
Unemployed	320 (96.10)	13 (3.90%)	1.97 (0.30–7.67)	0.389		
RRT status						
Negative	704 (94.37%)	42 (5.63%)		Ref		
Positive	119 (96.75%)	4 (3.25%)	1.77 (0.70–5.98)	0.281		

aOR = adjusted odds ratio; CI = confidence interval; NA = not available; Ref = reference; RRT = rostered routine test; STEM = science, technology, engineering, or medicine; uOR = unadjusted odds ratio. Bold *P*-values represent statistical significant variable.

* Adjusted odds ratio was controlled for significant variables such as age and flu vaccination status.

### Knowledge scores as a determinant of vaccination status.

After adjusting for demographic variables that were significantly different between the vaccinated and unvaccinated groups including age and flu vaccine status, high knowledge score was a significant determinant of the vaccinated group (aOR = 2.00, 95% CI: 1.09–3.68, *P* = 0.024; Supplemental Tables 1 and 2). In analysis of specific knowledge question items, participants who were less knowledgeable regarding the concept of herd immunity (aOR = 0.42, 95% CI: 0.022–0.81, *P* = 0.008), the suitability of the vaccine in people with known allergies (aOR = 0.14, 95% CI: 0.01–0.67, *P* = 0.055), and the perceived efficacy of Pfizer and Moderna in preventing symptomatic disease (aOR = 0.42, 95% CI: 0.22–0.81, *P* = 0.008) were negatively associated with the vaccinated group (Supplemental Tables 1 and 2).

### Attitudes score as a determinant of vaccination status.

After adjusting for demographic variables that were significantly different between the vaccinated and unvaccinated groups including age and flu vaccine status, it was found that individuals with a higher perceived efficacy of the vaccine (aOR = 1.19, 95% CI: 1.06–1.34, *P* = 0.004), perceived safety (aOR = 1.20, 95% CI: 1.06–1.36, *P* = 0.005), and willingness to uptake (aOR = 1.55, 95% CI: 1.30–1.85, *P* < 0.001) scores were significantly more likely to be vaccinated. Under the perceived efficacy section, individuals who perceive that it is impossible to reduce the incidence of COVID-19 without vaccination (aOR = 4.17, 95% CI: 2.08–8.07), *P* < 0.001) were more likely to be vaccinated. Under the perceived safety section, individuals who were confident in the vaccines offered by MOH (aOR = 3.43, 95% CI: 1.26–8.83), *P* = 0.013), unwaivered by possible side effects (aOR = 4.87, 95% CI: 2.45–10.23), *P* < 0.001), and motivated to encourage their friends and loved ones to receive the vaccine (unadjusted OR [uOR] = 8.31, 95% CI: 3.72–17.57), *P* < 0.001; aOR= 2.32, 95% CI: 0.81–6.23), *P* = 0.104) were more likely to be vaccinated. Additionally, individuals who stated that they would not delay vaccinations given that they are free (aOR = 15.30, 95% CI: 6.90–34.18), *P* < 0.001) and accessible (aOR = 4.01, 95% CI: 1.95–8.63), *P* < 0.001; Supplemental Tables 1 and 2) were also more often associated with the vaccinated group.

### Practice scores as a determinant of vaccination status.

There was no significant correlation between practice scores and COVID-19 vaccination status (uOR = 1.03, 95% CI: 0.80–1.29) *P* = 0.837) (Table 1). Across the vaccinated and unvaccinated groups, the most frequently practiced preventive activities included practicing social distancing measures regularly (94.82% and 91.30%, *P* = 0.269), sanitizing hands regularly (91.94% and 93.48%, *P* = 0.695), avoiding socializing in large groups (83.54% and 76.09%, *P* = 0.161), followed by avoiding staying in public areas for an extended period of time (79.17% and 78.26%, *P* = 0.876), not reusing disposable masks (66.05% and 65.22%, *P* = 0.902), and lastly washing hands using the seven steps of handwashing (51.09% and 58.70%, *P* = 0.289; Supplemental Tables 1 and 2).

### Practices before and after vaccination.

Among the vaccinated group, the only significant increase in preventive practices was washing hands using the seven steps of handwashing before and after vaccinations (50.37% and 52.36%, *P* = 0.008). There were no significant differences in the proportion of high practice scores (61.44% vs. 61.82%, *P* = 0.742) nor mean total score (4.67 ± 1.20 vs. 4.68 ± 1.19, *P* = 0.694) before and after vaccinations (Supplemental Table 3).

### Reasons against vaccination.

Among the unvaccinated group, reasons against uptakes were mainly to avoid the side effects (32.61%), preferring to take other measures to protect themselves (15.22%), and thinking vaccinations are not useful (10.87%). These were followed by not believing in vaccination (4.35%), believing that getting the COVID-19 infection is a better way of protecting themselves (2.17%), being afraid of needles (2.17%), and that vaccinations are against their personal beliefs (religious/ethical etc.; 2.17%).

### Inter-KAP differences.

There were statistically significant differences between the scores of knowledge and attitudes (*P* < 0.01, *r* = 0.132), attitudes and preventive practices before vaccination (*P* < 0.01, *r* = –0.095), and attitudes and practices after vaccination (*P* < 0.01, *r* = –0.130). However, the strength of the correlation between each of these sections are too small to suggest any meaningful implications (Table 1).

### Possible side effects from the vaccination.

A Fisher exact test was conducted between the vaccinated and nonvaccinated participants to determine whether the uptake of COVID-19 vaccination was a significant factor in the side effects experienced by individuals (Supplemental Table 4). Fever (*P* < 0.01), fatigue (*P* < 0.01), headache (*P* < 0.01), and muscle aches (*P* < 0.01) were shown to be statistically significant side effects caused by vaccination. On the other hand, rash (*P* = 2.580), shortness of breath (*P* = 0.250), stroke (*P* = 1.00), and heart attack (*P* = 1.00) were shown to be statistically insignificant. In general, individuals who were vaccinated were more likely to experience fever (aOR infinity, 95% CI: 11.37–infinity), *P* < 0.001), fatigue (aOR 10.43, 95% CI: 5.17–23.33, *P* < 0.001), headache (aOR 2.81, 95% CI: 1.43–6.06), *P* = 0.001), and muscle aches (aOR 7.68, 95% CI: 4.04–15.64), *P* < 0.001) after their vaccination.

### Characteristics and independent determinants of low knowledge scores.

There were 579 participants with a high knowledge score and 287 with a low knowledge score. A higher proportion of nonvaccinated participants (50.00% vs. 32.20%, *P* value = 0.013), annual flu vaccine nonrecipients (35.33% vs. 27.16%, *P* = 0.024), and those not on RRT (34.86% vs. 22.76%, *P* = 0.008) were in the low knowledge group compared with the high knowledge group. In terms of practices and attitudes scores, participants with lower perceived efficacy scores (11.25 ± 3.21 vs. 11.77 ± 3.22, *P* = 0.024), perceived safety scores (15.12 ± 2.99 vs. 15.93 ± 2.56, *P* < 0.001) and willingness to uptake scores (11.09 ± 2.24 vs. 11.51 ± 1.86, *P* = 0.006), were under the low knowledge group as compared with high knowledge group (Supplemental Table 5). From multivariable logistic regression analysis, only nonvaccinated participants (aOR = 1.75, *P* = 0.022) and lower perceived safety score (aOR = 1.07, *P* = 0.028) remain statistically correlated to low knowledge score group (Supplemental Table 5).

## DISCUSSION

This paper evaluates the KAP of Singaporean adults toward the COVID-19 vaccine. The survey was administered during a phase of the vaccination rollout where there were 3,448,193 (60.6%) individuals fully vaccinated as of August 1, 2021, resulting in a larger sample size of vaccinated individuals compared with nonvaccinated individuals.^26^

### Relationship between KAP and vaccine uptake.

A significant positive correlation between the knowledge score and the overall attitude scores of the survey respondents was observed. This indicates that high levels of knowledge partially influence vaccine attitudes and, subsequently, vaccine acceptance. A study conducted among Serbian medical students to access their knowledge and attitudes toward vaccination has seen a similar association between knowledge and attitude scores, which points toward a curriculum that addresses vaccination to be implemented within their medical schools.^27^

Nonsignificant correlation between knowledge and practice scores suggests that a high level of knowledge of COVID-19 vaccination is not sufficient to influence practices. These results were similar to the findings from a Ugandan study.^28^ The insufficient correlation between knowledge and practices suggests that knowledge alone is unlikely to convince individuals to adopt good practices.

A significant negative correlation between the attitude and practice scores could possibly be due to the influence of complacency and confidence in vaccine abundance, resulting in procrastination among individuals with a good attitude possibly because of the prolonged period of preventive practices in Singapore since the pandemic started.^29^

### High knowledge level of COVID-19 vaccine.

Overall, most respondents had a good knowledge of the vaccine, with 12 of 14 questions having accuracy rates above 50%. Similar to an earlier COVID-19 perception survey conducted among Singaporeans, a high knowledge score was observed.^30^ This could be due to effective educational campaigns and communication platforms implemented by the government with high levels of aid from media outlets, including educational videos, posters and advertisements on various official digital and traditional media platforms, engaging millions of Singaporeans.^5^^,^^31^ Such information campaigns have also been shown to be effective to correct misinformation.^32^

The high level of knowledge could be attributed to the level of education within our study population in which 92% of participants had at least a secondary school certification (Table 1). Scientific evidence-based policies and campaigns are instrumental in guiding a highly educated population to make informed decisions regarding the uptake of COVID-19 vaccine as a result of facts and knowledge being a prerequisite to base personal opinions, attitudes, and subsequent impact on health behaviours.^33^

#### Knowledge of herd immunity associated with positive vaccine uptake.

A significant proportion of vaccinated individuals understood that a large vaccinated population results in indirect protection for nonvaccinated individuals (e.g., individuals with weaker immune systems who cannot receive the vaccine). Response to this question was encouraging because it shows the success of current information campaigns in educating Singaporeans on the concept of herd immunity, in that to achieve this, large proportions of the population would have to be vaccinated.^7^ This correlation parallels studies conducted in the United Kingdom where the motivation to get vaccinated was related to the knowledge of herd immunity.^34^

Moreover, indirect success behind these information campaigns could be due to local government incentivization, where there was an understanding that increased vaccination rates would result in reduced COVID-19 related restrictions. The government assured Singaporeans that with at least 80% of the population taking up vaccination, Singapore would be more ready to move toward an endemic state with greater relaxation of COVID-19–related restrictions.^35^ This could thus pave the way for incentive-driven information campaigns on herd immunity knowledge, where incentives could therefore come in the form of scientifically backed relaxation of COVID-19 restriction policies, indirectly driving the vaccine uptakes.

#### Attitudes of vaccine uptake practices.

There is only moderate perceived efficacy of vaccine among participants. This suggests that the public understands that the COVID-19 vaccine does not confer full protection against the SARS-CoV-2 virus, and hence, despite vaccination, it is still of paramount importance to have other preventive measures such as mask wearing^36^ and proper handwashing^37^ to reduce COVID-19 transmission.

The public is generally favorable toward vaccine uptake, which is correlated with a high 94.67% of respondents being vaccinated. This can be attributed to a knowledgeable population, potentially influencing vaccine-acceptance and positive health behaviors. A similar trend of positive health KAP focusing on COVID-19 was also reported in a cross-sectional study in China, whereby the Chinese public similarly displayed strong knowledge, positive attitudes, and good practices toward containing the spread of COVID-19.^38^ The strict and swift top-down restriction measures from the government may also be partially attributable to this observation.^39^

However, in a similar cross-sectional study focusing on COVID-19 vaccines, adults in the United Kingdom have strong negative attitudes toward the vaccine.^40^ Distrust of vaccines and poor adherence to guidelines were reported. Factors associated with poor adherence to guidelines reported include low-income, female sex, and living with children. It was also hypothesized that income inequality, poor top-down educational campaigns, and low confidence in the government are possible reasons. This suggests that sociopolitical environment has a role in influencing vaccine uptake. Moreover, greater public–private partnership and community empowerment in the form of social responsibility advocacy would strongly encourage and incentivize citizens to get vaccinated, thus improving vaccine acceptance rates.^41^ Respectively, this includes the opportunity to ease certain social restrictions,^42^ engagement in low-risk outreach programs and numerous discounts for the vaccinated.^43^

#### Pro-vaccine and government groups associated with positive vaccine uptake.

Believing in the role of vaccination to reduce transmission, confidence in the government, and not being fearful of vaccine side effects were positively associated with COVID-19 vaccine uptake.

This study’s findings are consistent with a study conducted in Hong Kong, which also found that individuals who had greater acceptance of governmental COVID-19 preventive measures (indicating greater trust in government) and less concerns on vaccine safety were associated with higher willingness to vaccinate, and acceptance of governmental preventive measures was a strong predictor of vaccine acceptance. Reasons cited are that such individuals show greater confidence in the possibility of stopping COVID-19 community transmissions and are less worried and concerned about the disease.^44^ On the other hand, a Portuguese study found that factors contributing to vaccine hesitancy and refusal included low confidence in the COVID-19 vaccine and the health service response to the pandemic, as well as poor perception of government measures. This suggests that perceived vaccine efficacy and confidence in government are associated with vaccine uptake. Additionally, the Portuguese study also found a relationship between vaccine uptake and the release of information on the first vaccine’s safety and efficacy—vaccine refusal and delay was higher before the information release compared with after, suggesting that concern over vaccine side effects is another contributor toward vaccine uptake.^44^

This study also highlighted that people who declared high willingness to take up the COVID-19 vaccine do end up taking it. However, a Canadian study that surveyed university students found that although the majority of respondents were willing to take up COVID-19 vaccines, they still experienced the phenomenon of vaccine hesitancy due to perceived barriers such as perceived vaccine safety.^45^ This suggests that perceived vaccine safety may not be as predominant a perceived barrier among our study respondents, who were less likely to have been vaccinated against their wishes. This is likely due to the evidence-based communication approach used on a voluntary, opt-in basis; freely available and widely accessible vaccination information;^46^^,^^47^ and progressively convincing the fearful and doubtful subpopulation over time.

### Practices of Singaporeans before vaccination (nonstratified).

Regular social-distancing practices were the most frequently performed precautionary behavior. This was likely due to strict enforcement measures imposed by the government to reduce risk of transmission in Singapore. Deterrence measures used include media publicity of hefty fines imposed on offenders who breach COVID-19 regulations as well as recruitment of many safe-distancing ambassadors to enforce measures in public places.

In comparison, the least frequently performed practice was the seven steps of hand hygiene. This can be corroborated with a research study that showed that hand hygiene practices were inadequate and additional education efforts were needed to address this behavior.^48^ A cross-sectional survey on food and hand hygiene in Singapore identified a few key reasons for this inadequacy—namely, the busy working lifestyle among Singaporeans, a lack of effective programs to promote sustainable good hand hygiene practices, and complacencies among respondents.^49^ The complacency could stem from the perception of a low personal risk or belief that current behavior and practices were sufficient to reduce the likelihood of being exposed and/or infected with COVID-19. A study in Poland similarly found a positive correlation between handwashing practices, and perceived fear of contracting COVID-19 disease.^50^ The significance of hand hygiene practices for preventing the transmission of microorganisms and reducing the spread of infection should henceforth receive greater emphasis in future public health campaigns.

### Practices of Singaporeans after vaccination.

Our study showed that vaccinated individuals are more likely to continue to maintain the same high standards of safety practices as before vaccination. From a systemic review,^51^ when people collectively engage in preventive behaviors, including practicing personal hygiene and maintaining social distance, it is possible to control the spread of COVID-19. This study highlights that individual behaviors may dramatically decrease morbidity and mortality rates from COVID-19. Therefore, it is comforting that precautionary behaviors among the public are in progress to become the new status quo, even among the vaccinated, to reduce transmission of COVID-19.

### Perceived efficacy and safety scores: perceived vaccine safety a stronger influencing factor on willingness toward vaccine uptake.

Perceived efficacy and perceived safety scores—the other two aspects of attitudes our survey evaluated—were also found to be associated with positive uptake status. A recent study also observed improvement in Singaporeans’ attitudes toward COVID-19 vaccination due to waning concerns about vaccine safety and increase in confidence on vaccine efficacy (or perceived efficacy).^52^ These trends have also been reported in other high-income countries such as Kuwait.^53^

From a multinational study, it was found that, as a result of a decrease in influenza vaccine perception, there was a decrease in willingness to take the vaccines.^54^ Our study found that perceived safety was a stronger determinant of vaccine uptake compared with perceived efficacy.

This study found that respondents who took the flu vaccine had a better uptake attitude toward COVID-19 vaccines. This trend was also observed in the aforementioned Kuwait study, where respondents who took the flu vaccine in the last flu season, or even those who took it more than 1 year earlier, were generally more willing to take the COVID vaccine.^53^ Our study found that individuals who had previously taken the flu vaccine had generally higher perceived vaccine safety scores. It is henceforth highly suggestive that the positive association between flu vaccines and COVID-19 vaccine uptake attitudes is due to perceived vaccine safety.

Naturally, a portion of the population is skeptical of the safety of the vaccine and, should misinformation be unopposed, this will hinder its uptake. Such skepticism arises from distrust of the government, biomedical sciences, and/or Western medicine (specifically about vaccines).^54^ The sheer magnitude of vaccine-related information presented a wide range of perspectives—sometimes even opposing—from a multitude of sources (official and unofficial) can also trigger caution in people. Chat groups that act as echo chambers for thousands are one place where misinformation goes unregulated.^55^ Fortunately, the public’s knowledge regarding COVID-19 and the vaccine, based on scientific evidence and facilitated through well-established and coordinated communication platforms, potentially has enhanced trust in the government and health policies over time.^56^

## LIMITATIONS

### Limited sample size for respondents in the 60- to 89-year age group.

With only 10.08% of respondents in the 60 to 89 age range, a more comprehensive study targeting the elderly population in Singapore is necessary. Although there have been targeted campaigns toward the elderly,^57^ relatively poorer media literacy could limit these measures’ efficacy.^58^ A research study involving the elderly population in Singapore revealed both non–health- and health-related difficulty in Internet use,^59^ which signals room for improvement for holistic and inclusive approaches to defend the Singaporean population from COVID-19. Assuming weaker cognitive defenses against misinformation or traditional views of Western medicine and vaccines, these older adults are more vulnerable and prone to internalizing false information and making poor health decisions regarding the COVID-19 vaccine. With the high susceptibility of this population, especially to COVID-19, and growing mortality rates among them, studies focusing on the KAP of COVID-19 vaccine could thus be done among a cohort of individuals aged older than 60 in future. Increasing vaccine rates among this group is key to reduce the mortality associated with COVID-19 infections among the elderly.

### Limited sample size for nonvaccinated respondents.

The small sample size of the nonvaccinated population does not provide a reliable indication of the KAP of nonvaccinated individuals in Singapore, compared with the large sample size of vaccinated individuals. This could be attributed to the vaccination efforts made by the Singaporean government, resulting in an increasing proportion of the population being vaccinated. Another factor that should be considered is a nonresponse bias. A possible contributing factor could be the marginalization of nonvaccinated individuals.^60^ These individuals might choose not to do the survey, as reflected by the disproportionately low number of nonvaccinated respondents.

### Limited sample size for different racial demographics.

The proportion of racial demographics is heavily skewed toward the Chinese population, with a disproportionately large percentage of respondents being Chinese. This would not provide a good point of comparison for this demographic due to the low numbers (sample sizes < 50) of Malay, Indian, and Eurasian respondents.^61^ Future studies should be done to provide a more accurate indication of KAP for the various demographic groups of Singapore.

### Possible recall bias observed.

There might be a certain degree of recall bias present in the survey. This is exacerbated by the recall questions presented by questions in the practices (before vaccination) segment. Small details such as their use of the seven-step handwashing method would require some degree of recall, possibly giving rise to a recall bias. Future studies could potentially be done across two different time points (before and after vaccination) to reduce the incidence of a recall bias.

### Categorical bias within employment demographics.

A moderate degree of categorical bias might be seen for the employment demographic. This is due to the large variety of employment categories in the real-world context and the constraint of the study in listing these employment categories. This might result in some respondents having their employment type inaccurately categorized. In addition, the lack of clarity on what occupations were considered “high risk” made it hard to identify the occupations of respondents undergoing RRT. Future studies could potentially explore more on the occupational aspect of respondents for KAP toward the COVID-19 vaccine.

### Lack of evaluation on efficiency and effectiveness of elements of the government information campaign.

While our study indicates an overall success of the government’s information campaign, more analysis would be required to identify which mode of information dissemination is the most effective, for increased focus emphasized on those modes. This would allow for better use of resources and a more effective vaccination campaign.

## CONCLUSION

This study provides a robust understanding of the perception of COVID-19 vaccines in a high-income country, as well as the level of acceptance of Singapore adults toward infectious diseases guidelines. Governmental risk communication approaches have been useful in instilling high levels of vaccine knowledge. A positive attitude toward the vaccine was also observed among many Singaporeans, and, coupled with high vaccine knowledge, a high level of vaccination rates was achieved. This could be attributed to various factors, such as confidence in the government, good public health measures, and good delivery of information. However, measures can be improved to address specific areas, such as the general lack of proper handwashing practices and poor knowledge scores among older adults. This paves the way for more effective government measures to be designed to address the developing COVID-19 pandemic in areas such as vaccination uptake and public health practices.

## Supplemental files


Supplemental materials
