# Supplementary material for: Knowledge, Attitudes, and Practices of COVID-19 Vaccination among Adults in Singapore: A Cross-Sectional Study
Source: Am J Trop Med Hyg. 2022 Jul 18;107(3):540–50. doi: 10.4269/ajtmh.21-1259 (PMC9490657; doi:10.4269/ajtmh.21-1259)
Supplement: Supplementary file 1 [file tpmd211259.SD1.pdf]

Supplemental Table S1: Responses to Knowledge, Attitudes and Practices Sections

	Knowledge		Vaccinated group n(%) N = 823	Vaccinated group(%) N = 46	p-value
a	There are currently 3 approved COVID-19 vaccines in Singapore.	Correct	549 (66.95%)	28 (60.86)	0.423
		Not Correct	261 (33.05%)	18 (39.13%)	
›_asi	The COVID-19 vaccine provides protection against the COVID-19 virus by preventing symptomatic infections.	Correct	289 (35.23%)	19 (41.30%)	0.430
		Not Correct	531 (64.76%)	27 (58.70%)	
›_psi	The COVID-19 vaccine provides protection against the COVID-19 virus by preventing symptomatic infections.	Correct	523 (63.78%)	25 (54.35%)	0.211
		Not Correct	297 (36.22%)	21 (45.65%)	
b_rt	The COVID-19 vaccine provides protection against the COVID-19 virus by reducing transmission.	Correct	559 (68.17%)	26 (56.52%)	0.107
		Not Correct	261 (31.83%)	20 (43.48%)	
c	The COVID-19 vaccine can be administered to everyone, regardless of age.	Correct	707 (86.22%)	38 (82.61%)	0.511
		Not Correct	113 (13.78%)	8 (17.39%)	
d	Individuals with known allergies should not receive the vaccine unless evaluated to be suitable by a specialist.	Correct	116 (14.15%)	1 (2.17%)	**0.014**
		Not Correct	704 (85.85%)	45 (97.83%)	
e	There are no side effects at all from taking the COVID-19 vaccine.	Correct	761 (92.80%)	40 (86.96%)	0.147
		Not Correct	59 (7.20%)	6 (13.04%)	
f	Taking 1 dose of the COVID-19 vaccine is sufficient to gain immunity against COVID-19 virus.	Correct	753 (91.83%)	42 (91.30%)	0.785
		Not Correct	67 (8.17%)	4 (8.70%)	
g	Vaccinating a large proportion of the population results in indirect protection for non-vaccinated individuals (e.g. individuals	Correct	665 (81.10%)	28 (60.87%)	**0.002**
		Not Correct	155 (18.90%)	18 (39.13%)	

Supplemental Table S1: Responses to Knowledge, Attitudes and Practices Sections

	Knowledge		Vaccinated group n(%) N = 823	Vaccinated group(%) N = 46	p-value
**a**	There are currently 3 approved COVID-19 vaccines in Singapore.	Correct	549 (66.95%)	28 (60.86)	0.423
		Not Correct	261 (33.05%)	18 (39.13%)	
**b_asi**	The COVID-19 vaccine provides protection against the COVID-19 virus by preventing symptomatic infections.	Correct	289 (35.23%)	19 (41.30%)	0.430
		Not Correct	531 (64.76%)	27 (58.70%)	
**b_psi**	The COVID-19 vaccine provides protection against the COVID-19 virus by preventing symptomatic infections.	Correct	523 (63.78%)	25 (54.35%)	0.211
		Not Correct	297 (36.22%)	21 (45.65%)	
**b_rt**	The COVID-19 vaccine provides protection against the COVID-19 virus by reducing transmission.	Correct	559 (68.17%)	26 (56.52%)	0.107
		Not Correct	261 (31.83%)	20 (43.48%)	
**c**	The COVID-19 vaccine can be administered to everyone, regardless of age.	Correct	707 (86.22%)	38 (82.61%)	0.511
		Not Correct	113 (13.78%)	8 (17.39%)	
**d**	Individuals with known allergies should not receive the vaccine unless evaluated to be suitable by a specialist.	Correct	116 (14.15%)	1 (2.17%)	**0.014**
		Not Correct	704 (85.85%)	45 (97.83%)	
**e**	There are no side effects at all from taking the COVID-19 vaccine.	Correct	761 (92.80%)	40 (86.96%)	0.147
		Not Correct	59 (7.20%)	6 (13.04%)	
**f**	Taking 1 dose of the COVID-19 vaccine is sufficient to gain immunity against COVID-19 virus.	Correct	753 (91.83%)	42 (91.30%)	0.785
		Not Correct	67 (8.17%)	4 (8.70%)	
**g**	Vaccinating a large proportion of the population results in indirect protection for non-vaccinated individuals (e.g. individuals	Correct	665 (81.10%)	28 (60.87%)	**0.002**
		Not Correct	155 (18.90%)	18 (39.13%)	

	with weaker immune systems who cannot receive the vaccine).				
**h**	Antibodies against the COVID-19 virus are produced around 2 weeks after the first dose of the vaccine is administered.	Correct	553 (67.44%)	26 (56.52%)	0.147
		Not Correct	267 (32.56%)	20 (43.48%)	
**i**	COVID-19 vaccine is suitable even if you have serious allergic reactions in the past.	Correct	450 (54.88%)	28 (60.87%)	0.450
		Not Correct	370 (45.12%)	18 (39.13%)	
**j**	COVID-19 vaccine is suitable even if you have chronic medical conditions.	Correct	410 (50.00%)	17 (36.96%)	0.096
		Not Correct	410 (50.00%)	29 (63.04%)	
**k**	The Pfizer and Moderna vaccine's efficacy in preventing symptomatic COVID-19 disease is about 95%.	Correct	691 (84.27%)	32 (69.57%)	0.014
		Not Correct	129 (15.73%)	14 (30.43%)	
**l**	The Sinovac vaccine's efficacy is in preventing symptomatic COVID-19 disease about 50%.	Correct	466 (56.83%)	24 (52.17%)	0.545
		Not Correct	354 (43.17%)	22 (47.83%)	
**Total**			4.67 ± 3.39	6.03 ± 3.17	**0.011**
	Low		264 (32.30%)	23 (50.00%)	**0.016**
	High		556 (67.80%)	23 (50.00%)	

	**Attitudes**		**Vaccinated group (%) N = 823**	**Unvaccinated group (%) N = 46**	**p-value**
**Efficacy Section**			11.68 ± 3.22	10.17 ± 3.00	**0.002**
**a**	After receiving the vaccine, it is safe for me to go to crowded places more frequently.	Agree	186 (22.60%)	7 (15.22%)	0.503
		Neutral	115 (13.97%)	7 (15.22%)	
		Disagree	522 (63.43%)	22 (6.57%)	
**b**	After receiving the vaccine, it is safe for me to gather in large groups.	Agree	116 (14.09%)	3 (6.52%)	0.347
		Neutral	97 (11.79%)	6 (13.04%)	
		Disagree	610 (74.12%)	36 (80.43%)	

**c**	After receiving the vaccine, it is safe for me to not wear my mask in public.	Agree	35 (4.25%)	1 (2.17%)	0.404
		Neutral	51 (6.20%)	1 (2.17%)	
		Disagree	737 (89.55%)	44 (95.65%)	
**d**	After receiving the vaccine, it is safe for me to wash my hands less frequently.	Agree	30 (3.65%)	0	0.415
		Neutral	31 (3.77%)	2 (4.35%)	
		Disagree	762 (92.59%)	44 (95.65%)	
**g**	It is not possible to reduce the incidence of COVID-19 without vaccination.	Agree	611 (74.24%)	19 (41.30%)	**<0.001**
		Neutral	121 (14.70%)	12 (26.09%)	
		Disagree	91 (11.06%)	15 (32.61%)	
**Safety Section**			15.85 ± 2.57	12.35 ± 3.54	**<0.001**
**e**	I am confident in the COVID-19 vaccines offered by the Ministry of Health.	Agree	671 (81.53%)	18 (39.13%)	**<0.001**
		Neutral	121 (14.70%)	16 (34.78%)	
		Disagree	31 (3.77%)	12 (26.09%)	
**f**	**I** will encourage my family/friends/relatives to get vaccinated.	Agree	725 (88.09%)	18 (39.19%)	**<0.001**
		Neutral	68 (8.26%)	17 (36.96%)	
		Disagree	30 (3.65%)	11 (23.91%)	
**h**	The COVID-19 vaccine should be distributed fairly to all citizens and long-term pass holders.	Agree	722 (87.73%)	31 (67.39%)	<0.001
		Neutral	58 (7.05%)	12 (26.09%)	
		Disagree	43 (5.22%)	3 (6.52%)	
**i**	The side effects of the vaccine do not bother me.	Agree	396 (48.12%)	7 (15.22%)	<0.001
		Neutral	188 (22.84%)	7 (15.22%)	
		Disagree	239 (29.04%)	32 (69.57%)	
**Uptake Section**			11.52 ± 1.86	8.76 ± 2.57	**<0.001**
**j**	I will delay vaccination even if vaccination sites are accessible. (REVERSED)	Agree	727 (88.34%)	16 (34.78%)	**<0.001**
		Neutral	69 (8.38%)	10 (21.74%)	
		Disagree	27 (3.28%)	20 (43.48%)	
**k**	I will delay vaccination even if vaccination is free. (REVERSED)	Agree	745 (90.52%)	17 (36.96%)	**<0.001**
		Neutral	58 (7.05%)	10 (21.74%)	
		Disagree	20 (2.43%)	19 (41.30%)	

**l**	I am concerned about the availability of the vaccine.	Agree	240 (29.16%)	11 (23.91%)	0.159
		Neutral	216 (26.25%)	18 (39.13%)	
		Disagree	367 (44.95%)	17 (36.96%)	
	**Practices**		**Vaccinated group (%) N = 823**	**Unvaccinated group (%) N = 46**	**p-value**
**a**	Do you avoid socialising in large groups?	No	132 (16.10%)	11 (23.91%)	0.157
		Yes	688 (83.90%)	35 (76.09%)	
**b**	Do you avoid staying in public areas for an extended period of time?	No	171 (20.85%)	10 (31.74%)	0.853
		Yes	649 (79.15%)	36 (78.26%)	
**c**	Do you wash hands using the 7 steps of handwashing?	No	406 (49.51%)	19 (41.30%)	0.363
		Yes	414 (50.49%)	27 (58.70%)	
**d**	Do you reuse disposable masks? (REVERSED)	No	276 (50.49%)	16 (34.78%)	0.874
		Yes	544 (66.34%)	30 (65.33%)	
**e**	Do you wash/sanitise your hands regularly?	No	67 (8.17%)	3 (6.52%)	1
		Yes	753 (91.83%)	43 (93.48%)	
**f**	Do you practice social distancing measures regularly?	No	41 (5.00%)	4 (8.70%)	0.291
		Yes	779 (95.00%)	42 (91.30%)	
**Total**			4.67 ± 1.45	4.63 ± 1.45	0.863
	Low		315 (38.28%)	17 (36.96%)	1.00
	High		508 (61.73%)	29 (63.04%)	

Supplemental Table S2: Univariate and Multivariable Regression of KAP Domains to Vaccination Status

Knowledge Items		UnAdj OR [95% CI]	p-value	Adj OR* [95% CI]	p-value
a	Correct		REF		
	Not Correct	0.77 [0.42, 1.44]	0.396		
b_asi	Correct		REF		
	Not Correct	1.29 [0.70, 2.35]	0.405		
b_psi	Correct		REF		
	Not Correct	0.68 [0.37, 1.24]	0.199		
b_rt	Correct		REF		
	Not Correct	0.61 [0.33, 1.12]	0.104		
c	Correct		REF		
	Not Correct	0.76 [0.36, 1.79]	0.493		
d	Correct		REF		REF
	Not Correct	0.13 [0.01, 0.63]	**0.049**	0.14 [0.01, 0.67]	0.055
e	Correct		REF		
	Not Correct	0.52 [0.23, 1.40]	0.150		
f	Correct		REF		
	Not Correct	0.93 [0.36, 3.18]	0.900		
g	Correct		REF		REF
	Not Correct	0.36 [0.20, 0.68]	**0.001**	0.42 [0.22, 0.81]	**0.008**

h	Correct		REF		
	Not Correct	0.63 [0.35, 1.16]	0.129		
i	Correct		REF		
	Not Correct	1.28 [0.70, 2.39]	0.427		
j	Correct		REF		
	Not Correct	0.59 [0.31, 1.07]	0.088		
k	Correct		REF		REF
	Not Correct	0.43 [0.23, 0.85]	**0.011**	0.54 [0.28, 1.10]	0.080
l	Correct		REF		
	Not Correct	0.83 [0.46, 1.51]	0.536		
**Knowledge Score High/Low**		**UnAdj OR [95% CI]**	**p-value**	**Adj OR [95% CI]**	**p-value**
**Low**			REF		REF
**High**		2.11 [1.16, 3.84]	**0.014**	2.00 [1.09, 3.68]	**0.024**
**Attitudes: Efficacy Subsection**		**UnAdj OR [95% CI]**	**p-value**	**Adj OR [95% CI]**	**p-value**
a	Agree		REF		
	Neutral	0.62 [0.21, 1.85]	0.380		
	Disagree	0.61 [0.25, 1.34]	0.252		
b	Agree		REF		
	Neutral	0.42 [0.09, 1.63]	0.226		
	Disagree	0.43	0.161		

		[0.10, 1.20]			
c	Agree		REF		
	Neutral	1.46 [0.06, 37.68]	0.792		
	Disagree	0.48 [0.03, 2.30]	0.473		
d	Agree		REF		
	Neutral	NA	0.984		
	Disagree	NA	0.984		
g	Agree		REF		REF
	Neutral	0.31 [0.15, 0.68]	**0.002**	0.32 [0.15, 0.69]	**0.003**
	Disagree	0.19 [0.09, 1.34]	**<0.001**	0.18 [0.08, 0.37]	**<0.001**
**Attitudes: Safety Subsection**		**UnAdj OR [95% CI]**	**p-value**	**Adj OR [95% CI]**	**p-value**
e	Agree		REF		REF
	Neutral	0.20 [0.10, 0.41]	**<0.001**	0.62 [0.25, 1.56]	0.302
	Disagree	0.07 [0.03, 0.16]	**0.002**	0.39 [0.12, 1.32]	0.124
f	Agree		REF		REF
	Neutral	0.10 [0.05, 0.20]	**<0.001**	0.22 [0.09, 0.54]	**0.001**
	Disagree	0.07 [0.03, 0.16]	**<0.001**	0.21 [0.07, 0.71]	**0.011**
h	Agree		REF		REF
	Neutral	0.21 [0.10, 0.44]	**<0.001**	0.46 [0.20, 1.14]	0.082
	Disagree	0.62 [0.21, 2.63]	0.437	1.84 [0.52, 9.01]	0.391
	Agree		REF		REF

i	Neutral	0.15 [0.07, 0.36]	**<0.001**	2.45 [0.68, 11.74]	0.202
	Disagree	0.03 [0.01, 0.05]	**<0.001**	0.38 [0.15, 0.87]	**0.025**
**Attitudes: Uptake Subsection**		**UnAdj OR [95% CI]**	**p-value**	**Adj OR [95% CI]**	**p-value**
j	Agree		REF		REF
	Neutral	0.15 [0.07, 0.36]	**<0.001**	0.42 [0.09, 2.77]	0.331
	Disagree	0.03 [0.01, 0.06]	**<0.001**	0.18 [0.03, 1.50]	0.084
k	Agree		REF		REF
	Neutral	0.13 [0.06, 0.31]	**<0.001**	0.31 [0.05, 1.54]	0.188
	Disagree	0.02 [0.01, 0.05]	**<0.001**	0.13 [0.02, 0.82]	**0.045**
l	Agree		REF		
	Neutral	0.55 [0.25, 1.17]	0.129		
	Disagree	0.99 [0.44, 2.13]	0.979		
**Attitudes: Subsection Scores**		**UnAdj OR [95% CI]**	**p-value**	**Adj OR [95% CI]**	**p-value**
Efficacy Score		1.20 [1.07, 1.34]	**0.002**	1.19 [1.06, 1.34]	**0.004**
Safety Score		1.45 [0.42, 6.47]	**<0.001**	1.20 [1.06, 1.36]	**0.005**
Uptake Score		1.81 [1.57, 2.10]	**<0.001**	1.55 [1.30, 1.85]	**<0.001**
**Practices**		**UnAdj OR [95% CI]**	**p-value**	**Adj OR [95% CI]**	**p-value**
No			REF		

**a**	Yes	1.65 [0.78, 3.22]	0.165		
	No		REF		
**b**	Yes	1.06 [0.49, 2.10]	0.876		
	No		REF		
**c**	Yes	0.72 [0.39, 1.31]	0.291		
	No		REF		
**d**	Yes	1.04 [0.55, 1.91]	0.902		
	No		REF		
**e**	Yes	0.79 [0.19, 2.23]	0.695		
	No		REF		
**f**	Yes	1.82 [0.53, 4.77]	0.275		
**Practice Scores**		**UnAdj OR [95% CI]**	**p-value**	**Adj OR [95% CI]**	**p-value**
**Low**			REF		
**High**		0.95 [0.50, 1.73]	0.858		

*Adjusted OR was controlled for significant variables such as age and flu vaccination status

Supplemental Table S3: Comparison of COVID-19 regulation practices before and after vaccination

Practices Question Item	Before vaccination n(%) N=804	After vaccination n(%) N=804	p-value
**Do you avoid socialising in large groups?**			
No	128 (15.92%)	129 (16.04%)	0.908
Yes	676 (84.08%)	675 (83.96%)	
**Do you avoid staying in public areas for an extended period of time?**			
No	167 (20.77%)	179 (22.26%)	0.185
Yes	637 (79.23%)	625 (77.74%)	
**Do you wash hands using the 7 steps of handwashing?**			
No	399 (49.63%)	383 (47.64)	**0.008**
Yes	405 (50.37%)	421 (52.36)	
**Do you reuse disposable masks?**			
No	274 (34.08%)	273 (33.96%)	0.876
Yes	530 (65.92%)	531 (66.04%)	
**Do you wash/sanitise your hands regularly?**			
No	66 (8.21%)	68 (8.46%)	0.683
Yes	738 (91.79%)	736 (91.54%)	
**Do you practice social distancing measures regularly?**			
No	39 (4.85%)	33 (4.10%)	0.201
Yes	765 (95.15%)	771 (95.90%)	
**High/Low Category**			
Low (0 to 4)	310 (38.56%)	307 (38.18%)	0.742
High (5 to 6)	494 (61.44%)	497 (61.82%)	
**Total Practice Score (0 to 6)**			
Mean ± SD	4.67 ± 1.20	4.68 ± 1.19	0.694

Supplemental Table S4: Incidence of individuals experiencing side effects within the last 14 days

Side effect	Vaccinated n(%) N=804	Unvaccinated n(%) N=65	p-value	Adj OR (95% CI)
**Fever / Chills**	323 (40.17%)	0 (0.00%)	**<0.001**	N.A.
**Fatigue**	527 (65.55%)	10 (15.38%)	**<0.001**	10.43 [5.17, 23.33]
**Headache**	293 (36.44%)	11 (16.92%)	**0.001**	2.81 [1.43, 6.06]
**Muscle Aches**	529 (65.80%)	13 (20.00%)	**<0.001**	7.68 [4.04, 15.64)
**Rash**	61 (7.59%)	2 (3.08%)	0.220	2.58 [0.66, 22.32]
**Pain, Redness or Swelling**	426 (52.99%)	N.A.	N.A.	N.A
**SOB**	45 (5.60%)	1 (1.54%)	0.246	3.79 [0.62, 155.44]
**Stroke**	5 (0.62%)	0 (0.00%)	1	N.A.
**Heart Attack**	5 (0.62%)	0 (0.00%)	1	N.A.

Supplemental Table S5: Univariate and multivariable logistic regression analysis for low knowledge score

		Low Score n(%) N=287	High Score n(%) N=579	UnAdj OR (95% CI)	p-value	Adj OR* (95% CI)	p-value
**Vaccination Status**							
	Non-vaccinated	23 (50.00%)	23 (50.00%)		REF		REF
	Vaccinated	264 (32.20%)	556 (67.80%)	2.11 [1.16, 3.84]	**0.014**	0.67 [0.22, 2.05]	0.483
**Age**							
	21 - 29	90 (30.72%)	203 (69.28%)		REF		REF
	30 - 39	26 (36.63)	45 (63.38%)	0.77 [0.44, 1.33]	0.339	1.00 [0.57, 1.78]	0.998
	40 - 49	45 (31.25%)	99 (68.75%)	0.98 [0.64, 1.51]	0.910	1.27 [0.80, 2.02]	0.319
	50 - 59	86 (31.73%)	185 (68.27%)	0.95 [0.67, 1.36]	0.794	1.13 [0.77, 1.65]	0.535
	≥ 60	40 (45.98%)	47 (54.02%)	0.55 [0.32, 0.85]	**0.009**	0.61 [0.36, 1.01]	0.055
**Race**							
	Chinese	270 (33.37%)	539 (66.63%)		REF		
	Malay	4 (30.77%)	9 (69.23%)	1.13 [0.36, 4.19]	0.843		
	Indian	9 (37.50%)	15 (62.50%)	0.83 [0.37, 2.01]	0.673		
	Eurasian	2 (40.00%)	3 (60.00%)	0.75 [0.12, 5.73]	0.755		
	Others	2 (13.33%)	13 (86.67%)	3.26 [0.89, 20.92]	0.122		
**Sex**							
	Female	169 (32.63%)	349 (67.37%)		REF		
	Male	118 (33.91%)	230 (66.09%)	0.94	0.694		

			[0.71, 1.26]			
**Marital Status**						
Married	163 (33.13%)	329 (66.87%)		REF		
Unmarried	124 (33.16%)	250 (66.84%)	1.00 [0.75, 1.33]	0.994		
**Education Level**						
None	1 (100.00%)	0 (0.00%)		REF		
Primary	0 (0.00%)	1 (100.00%)	N.A.	0.971		
Secondary	15 (41.67%)	21 (58.33%)	N.A.	0.979		
Pre-University	77 (33.33%)	154 (66.67%)	N.A.	0.979		
University and Above	183 (32.38%)	384 (67.72%)	N.A.	0.979		
Others	11 (36.67%)	19 (63.33%)	N.A.	0.979		

	Low Score n(%) N=587	High Score n(%) N=579	UnAdj OR (95% CI)	p-value	Adj OR* (95% CI)	p-value
**Income Level ($)**						
< 1,000	11 (27.50%)	29 (72.50%)		REF		
1,000 - 4,000	32 (34.41%)	61 (65.59%)	0.72 [0.31, 1.60]	0.436		
4,000 - 7,000	49 (38.58%)	78 (61.42%)	0.60 [0.27, 1.29]	0.205		
7,000 - 10,000	44 (28.03%)	113 (71.97%)	0.97 [0.43, 2.07]	0.947		
> 10,000	151 (33.63%)	298 (66.37%)	0.75 [0.35, 1.50]	0.431		
**Flu Vaccine**						
No	224 (35.33%)	410 (64.67%)		REF		REF
Yes	63 (27.16%)	169 (72.84%)	1.47 [1.06, 2.05]	**0.024**	1.30 [0.92, 1.84]	0.135
**Medical Conditions**						
None	239 (32.97%)	486 (67.03%)		REF		

³ 1	48 (34.04%)	93 (65.96%)	0.95 [0.65, 1.40]	0.804		

	Low Score n(%) N=278	High Score n(%) N=579	UnAdj OR (95% CI)	p-value	Adj OR* (95% CI)	p-value
**Employment Sector**						
Commerce (Retail and Trade)	10 (37.04%)	17 (62.96%)		REF		
Community, Social and Personal Services	18 (36.00%)	32 (64.00%)	1.05 [0.39, 2.75]	0.928		
Education	22 (35.48%)	40 (64.52%)	1.07 [0.41, 2.71]	0.888		
Finance and Business	53 (37.86%)	87 (62.14%)	0.97 [0.40, 2.23]	0.936		
Food and Beverages	1 (8.33%)	11 (91.67%)	6.47 [1.01, 127.34]	0.095		
Hotels and Tourism	2 (50.00%)	2 (50.00%)	0.59 [0.06, 5.53]	0.622		
Others	45 (41.28%)	64 (58.72%)	0.84 [0.34, 1.97]	0.688		
STEM and Healthcare	30 (31.25%)	66 (68.75%)	1.29 [0.52, 3.13]	0.571		
Transport, Storage and Communications	12 (34.29%)	23 (65.71%)	1.13 [0.39, 3.23]	0.822		
Unemployed	94 (28.40%)	237 (71.60%)	1.48	0.344		

			[0.63, 3.31]			
**RRT Status**						
Negative	259 (34.86%)	484 (65.14%)		REF		REF
Positive	28 (22.76%)	95 (77.24%)	1.82 [1.18, 2.89]	**0.009**	1.75 [1.10, 2.87]	**0.022**
**Perceived Efficacy Score (Mean ± SD)**	11.25 ± 3.21	11.77 ± 3.22	1.05 [1.01, 1.10]	**0.025**	1.05 [1.00, 1.10]	0.062
**Perceived Safety Score (Mean ± SD)**	15.11 ± 2.99	15.93 ± 2.56	1.11 [1.06, 1.17]	**<0.001**	1.07 [1.01, 1.14]	**0.028**
**Willingness to Uptake Score (Mean ± SD)**	11.09 ± 2.24	11.51 ± 1.86	1.11 [1.03, 1.19]	**0.003**	1.05 [0.97, 1.15]	0.231
**Practices (Bef) Score**						
Low	117 (35.24%)	215 (64.76%)		REF		
High	170 (31.84%)	364 (68.16%)	1.08 [0.87, 1.56]	0.217		

*Adjusted for age, flu vaccine, covid vaccine, vaccination status, RRT, efficacy score, safety score and uptake score.
